# Preparation of Well-Defined Propargyl-Terminated Tetra-Arm Poly(*N*-isopropylacrylamide)s and Their Click Hydrogels Crosslinked with β-cyclodextrin

**DOI:** 10.3390/polym8040093

**Published:** 2016-03-28

**Authors:** Jianquan Wang, Zhe Zhu, Xin Jin, Zhujun Li, Yizhen Shao, Ziqiang Shao

**Affiliations:** Beijing Engineering Research Center of Cellulose and Its Derivatives, School of Materials Science and Engineering, Beijing Institute of Technology, Beijing 100081, China; jxzhuzhe@163.com (Z.Z.); erinkim@sina.com (X.J.); 1120132214@bit.edu.cn (Z.L.); syzsyz1_1@163.com (Y.S.); shaoziqiang@263.net (Z.S.)

**Keywords:** *N*-isopropylacrylamide, β-cyclodextrin, click chemistry, temperature sensitivity, hydrogel

## Abstract

As an important class of reversible deactivation radical polymerization (RDRP), reversible addition fragmentation chain transfer (RAFT) polymerization has attracted great attention attributed to its facile and flexible features to prepare well-defined polymers with different complex structures. In addition, the combination of RAFT with click chemistry provides more effective strategies to fabricate advanced functional materials. In this work, a series of temperature responsive tetra-arm telechelic poly(*N*-isopropylacrylamide)s (PNIPAs) with propargyl end groups were prepared for the first time through RAFT and subsequent aminolysis/Michael addition modification. The temperature sensitivities of their aqueous solutions were researched via turbidity measurement. It was found that the phase transition temperature of obtained PNIPAs increased with their molecular weights ascribed to their distinctions in the hydrophobic/hydrophilic balance. Subsequently, β-cyclodextrin (β-CD) functionalized with azide moieties was used to crosslink the prepared propargyl-terminated tetra-arm PNIPAs through click chemistry, fabricating corresponding hydrogels with thermoresponse. Similar to their precursors, the hydrogels demonstrated the same dependence of volume phase transition temperature (VPTT) on their molecular weights. In addition, the incorporation of β-CD and the residual groups besides crosslinking may provide a platform for imparting additional functions such as inclusion and adsorption as well as further functionalization.

## 1. Introduction

Recently, the development of reversible deactivation radical polymerization (RDRP) has opened a series of effective routes to prepare well-defined polymers with various advanced architectures and functionalities. Generally, the most frequently applied RDRP techniques include atom transfer radical polymerization (ATRP) [1], nitroxide mediated radical polymerization (NMP) [2], and reversible addition fragmentation chain transfer (RAFT) polymerization [3]. In virtue of these methods, the design of initiators and chain transfer agents (CTAs) as well as post-polymerization modification supplies the basis for tailoring the structure and functionality of a certain macromolecule. In the three RDRP strategies, RAFT polymerization may be the most versatile one due to its broad applicability to the majority of monomers subject to conventional radical polymerization and the diversity of functionalization [3,4]. As reviewed in literatures [4,5,6], the utilization of RAFT CTA provides researchers with abundant imagination containing CTA structure design and/or thiocarbonylthio transformation and subsequent thio-chemistry.

It is well-known that *N*-isopropylacrylamide (NIPA) is a typical monomer for synthesizing temperature responsive polymers, and the aqueous solution of NIPA homopolymer has a lower critical solution temperature (LCST) around 32 °C, below and above which the polymer will dissolve and precipitate, respectively. NIPA based polymer materials have been extensively studied because of their wide potential applications in biomaterials, separation, sensors, actuators, *etc.* [7,8,9]. A variety of NIPA based polymers with different structures such as linear, branched, cyclic, brush-like, and so on, have been successfully prepared through RAFT polymerization with or without combining other methods [10,11,12,13,14]. The adopted strategies consist of the choice of CTA compounds and/or further modification of end groups. Lv and coworkers grafted PNIPA chains onto dextran backbones to get comb-like copolymers using a dodecylthiocarbonothioylthio grafted dextran as a RAFT agent, which could assemble into temperature sensitive nanogels in water through noncovalent interactions; after aminolysis, the nanogels were endowed with redox sensitivity besides their inherent thermo sensitivity due to the generation of thiols and the formation of disulfide [15]. Thermoresponsive gold nanoparticles were synthesized through RAFT polymerization of NIPA and subsequent reduction of dithioester to thiol end groups by sodium borohydride, followed by conjugation with Au nanoparticles [16]. Huang *et al.* conjugated maleimide ended PNIPA with protein, obtaining diblock copolymers able to be used as highly active nanostructured biocatalysts [17].

Click chemistry is another powerful method to fabricate well-defined functional and/or complex polymer materials. Azide-alkyne cycloaddition (AAC), thiol-ene addition, and Diels–Alder cycloaddition are three typical click reactions. As reviewed in detail by Harvison and Lowe [4], the marriage of RAFT and click chemistry has yielded fruitful results, and a number of novel polymer materials with desirable structures and properties have been derived. Here, some NIPA-based materials are taken as examples. Amphiphilic “tadpole” block copolymers with cyclic polystyrene as “head” and PNIPA as “tail”, respectively, were made via RAFT and Cu-catalyzed AAC (CuAAC) [18]. Various telechelic PNIPAs were obtained through aminolysis of thioester groups and subsequent thiol-ene Michael addition of the resulting thiols to (meth)acrylates in near quantitative yields [19,20]. In addition, different well-defined PNIPA-based hydrogels were also constructed in succession via combined use of RAFT and click chemistry. Ooi *et al.* prepared linear telechelic azido-ended PNIPAs applying a bis azido-RAFT agent, followed by CuAAC with a tetra-acetylene crosslinker, obtaining highly regular PNIPA networks [21]. Then, they explored photo initiated thiol-ene addition to build a PNIPA “model network” using a tetra-arm mercapto propionate molecule to crosslink an α,ω-bis vinyl PNIPA modified from a RAFT PNIPA [22]. Pasale and coworkers fabricated multi-responsive hyaluronan-PNIPA hybrid hydrogels using azido-grafted hyaluronic acid and α,ω-bis propargyl end-capped PNIPAs, which were derived from mono-alkyne ended RAFT PNIPAs and further conversion of another end to alkyne [23]. We have ever prepared a well-defined linear PNIPA carrying pendant azido groups using RAFT polymerization and the following modification, which was click-crosslinked with multi-alkynyl terminated small molecules to get well-defined PNIPA hydrogels [24]. Furthermore, the same linear azido-PNIPA was crosslinked with several α,ω-bis propargyl PNIPAs with different molecular weights that were synthesized using a bis propargyl-RAFT agent, tailoring a series of click hydrogels with tunable properties [25].

**Scheme 1:**
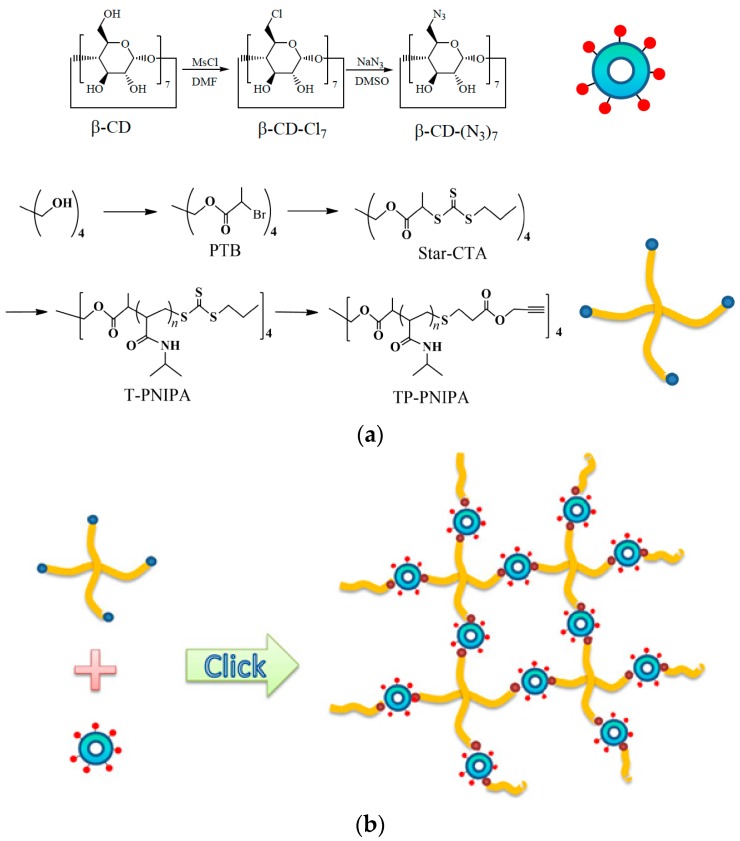
Synthetic routes of functional polymers and hydrogels thereof. (**a**) Synthesis of small- and macro-molecules; (**b**) Hydrogel fabrication through click chemistry.

As for the aforementioned PNIPA hydrogels, the advantage is that their properties could be finely regulated by adjusting some structural parameters of used materials because of their well-defined nature, and another common feature of used PNIPAs is α,ω-bis functionalization of linear polymers. In this work, we focus on the synthesis of tetra-functionalized PNIPAs with the chain extension from a pentaerythritol derivative via RAFT polymerization and following end-group modification to tetra-propargyl ones through aminolysis/Michael addition one-pot strategy. Next, the obtained tetra-propargyl terminal PNIPAs are used to construct well-defined hydrogels applying CuAAC click chemistry, with a β-cyclodextrin (β-CD) azido-derivative as crosslinker. The detailed synthetic routes are shown in Scheme 1. Here, β-CD is chosen in consideration of its structure with hydrophilic outer surface and lipophilic central cavity, as well as its performance for inclusion and adsorption to some substances, which makes it find potential applications in the fields of adsorbent, drug delivery and so on. The advantage of this research is the conjugation of smart PNIPA with β-CD via highly controllable RAFT and click chemistry to get well-defined structures, which is very important for investigating structure-property relationship more accurately from the viewpoint of fundamental respect. On the other hand, this system may find its application potentials in some areas such as intelligent separation and delivery. The synthesis of tetra-functionalized PNIPAs, their thermo-sensitivities in aqueous solutions, and the characteristics of obtained click hydrogels are the emphases of this study. Further investigations using the prepared hydrogels as platforms will be performed in future.

## 2. Materials and Methods

### 2.1. Materials

2-Bromopropionyl bromide, 1-propanethiol, propargyl acrylate (PA), and tris(2-carboxyethyl)phosphine hydrochloride (TCEP–HCl) were bought from Alfa Aesar China (Tianjin, China) Co., Ltd. *N*-isopropylacrylamide (NIPA) was purchased from TCI (Shanghai, China) Development Co., Ltd. β-cyclodextrin (β-CD) and 2, 2′-Azobis(isobutyronitrile) (AIBN) were supplied by Shanghai Jingchun Biotech Co. (Shanghai, China). Other chemicals (AR grade) are commercial goods of Beijing Chemicals Co. (Beijing, China). Prior to use, NIPA and AIBN were recrystallized from hexane and ethanol, respectively; tetrahydrofuran (THF) was distilled for RAFT polymerization. All other reagents were used as received.

### 2.2. Preparation of β-CD-(N_3_)_7_

β-CD derivative with seven azido groups on the 6-position of every glucose unit was produced with reference to the literature [26,27]. Firstly, dried β-CD (2.27 g, 2 mmol) was reacted with methyl sulfonyl chloride (8.34 g, 72.8 mmol) in 10 mL of dry DMF at 65 °C for 4 day. Then, 50 mL of 2 M Na_2_CO_3_ solution was added dropwise to the reaction mixture under stirring, and a great deal of brown milky suspension was generated, which was diluted into 200 mL. The suspension was centrifuged and washed for 4 cycles until pH reached a neutral level. The obtained muddy product was dried and 2.32 g of β-CD-Cl_7_ was recovered, with a yield of 92 %. Its ^1^H and ^13^C NMR spectra are shown in Figure S1 (Supplementary Material). ^1^H NMR (DMSO-*d*_6_), *δ* (ppm): 5.9 (1H, –O*H* adjacent to 2-position), 5.8 (1H, –O*H* linked to 3-position), 4.9 (1H, 1-position), 4.1 (1H, 5-position) 3.8 (2H, 3- and 4-position), 3.6 (1H, 2-position), 3.28–3.45 (2H, 6-position, overlapped by H_2_O). ^13^C NMR (DMSO-*d*_6_), *δ* (ppm): 102.8 (1-C), 83.0 (4-C), 72.5 (3-C), 71.8 (2-C), 70.83 (5-C), 45.6 (6-C). Elemental analysis: C 37.68 (cal. 39.88), H 5.20 (cal. 4.99).

Subsequently, β-CD-Cl_7_ (1 g, 0.8 mmol) was azidized with NaN_3_ (1.1 g, 16.8 mmol) in 3 mL DMSO at 80 °C for 36 h, and the resulting solution was poured into 50 mL of water to get muddy suspension, which was subjected to centrifugation and washed for 3 cycles. Finally, 0.85 g (yield 85 %) of grey solids, β-CD-(N_3_)_7_ was obtained after drying in a vacuum. And its ^1^H and ^13^C NMR spectra are shown in Figure S2 (Supplementary Material). ^1^H NMR (DMSO-*d*_6_), *δ* (ppm): 5.9 (1H, –O*H* adjacent to 2-position carbon), 5.8 (1H, –O*H* linked to 3-position carbon), 4.9 (1H, 1-position), 3.7–3.8 (2H, 3- and 4-position), 3.6 (2H, 2- and 5-position), 3.32–3.34 (2H, 6-position, overlapped by H_2_O). ^13^C NMR (DMSO-*d*_6_), *δ* (ppm): 102.5 (1-C), 83.6 (4-C), 73.1 (3-C), 72.5 (2-C), 70.8 (5-C), 51.8 (6-C). Elemental analysis: C 36.98 (cal. 38.47), H 5.08 (cal. 4.81), N 21.67 (cal. 22.44).

### 2.3. Synthesis of Star-CTA

Tetra-functionalized star-CTA was synthesized according to the literature [28]. Firstly, pentaerythritol tetrakis(2-bromopropionate) (PTB) was prepared. In detail, pentaerythritol (5.0 g, 37 mmol), triethylamine (16.2 g, 160 mmol), and THF (160 mL), which were dried in advance, were charged into a 500 mL three-necked round-bottom flask in an ice bath, and the mixture was purged with N_2_ under stirring. After 10 min, a solution of 2-bromopropionyl bromide (34.5 g, 160 mmol) in 66 mL of THF was dropped into the above reaction system through a constant-voltage funnel. The reactant mixture was left to stir at room temperature for 12 h under N_2_ protection. Afterwards, the resulting mixture was filtered to remove precipitate before THF removal via rotary evaporation. Then, the residuum was dissolved in 30 mL of ethyl acetate, which was washed with 6 wt % NaHCO_3_ solution (50 mL × 3) and distilled water (100 mL × 3), and dried over anhydrous Na_2_SO_4_. Then, the solvent was rotary-evaporated to get white powder, which was further recrystallized from *n*-hexane twice, obtaining 6.3 g of white crystal, yield = 26%. ^1^H NMR (CDCl_3_), *δ* (ppm): 1.74 (12H, –CH(C*H*_3_)–Br), 4.14 (4H, –C*H*(CH_3_)–Br), 4.29 (8H, C–C*H*_2_–O–). ^13^C NMR (CDCl_3_), *δ* (ppm): 169.5 (–*C*=O), 62.9 (–C–*C*H_2_O–), 43.0 (–*C*–CH_2_–), 39.2 (–*C*H(CH_3_)–Br), 21.6 (–*C*H_3_). Elemental analysis: C 28.87 (cal. 30.18), H 3.78 (cal. 3.55).

A mixture of CS_2_ (5.5 g, 72 mmol) in 60 mL of THF was added dropwise into the solution of triethylamine (7.3 g, 72 mmol), 1-propanethiol (5.5 g, 72 mmol) and THF (160 mL) in a 500 mL three-necked round-bottom flask immersed in an ice bath. Then, the system was stirred at 0 °C for 1 h and at room temperature for another 1 h. Afterwards, it was placed into an ice bath again and purged with N_2_ for 20 min. A solution of PTB (6.1 g, 9 mmol) in 60 mL of THF was dropped into the above reaction system at 0 °C within 30 min. The reaction mixture was stirred for 24 h and, during that time, it was allowed to warm up to room temperature. The obtained mixture was rotary-evaporated to remove solvent and then was redissolved in 200 mL of dichloromethane (DCM), followed by washing with 10 wt % HCl solution (100 mL × 3) and distilled water (100 mL × 3), and drying with anhydrous Na_2_SO_4_. After the evaporation of DCM, the crude product was subjected to column chromatography separation in silica gel by the method of gradient elution using ethyl acetate and petroleum ether mixtures as eluents (ethyl acetate/petroleum ether: 1/30→1/20→1/10). Finally, 6.5 g of yellow oil product nominated as star-CTA was obtained, yield = 75%. ^1^H NMR (CDCl_3_), *δ* (ppm): 1.04 (12H, –CH_2_–CH_2_–C*H*_3_), 1.60 (12H, –C(O)–C(C*H*_3_)–S), 1.75 (8H, –CH_2_–C*H*_2_–CH_3_), 3.36 (8H, –C*H*_2_–CH_2_–CH_3_), 4.09 (8H, –C–C*H*_2_–O–), 4.84 (4H, –C*H*(CH_3_)–S–). ^13^C NMR (CDCl_3_), *δ* (ppm): 220.6 (*C*=S), 170.5 (*C*=O), 62.5 (–C*C*H_2_–O–), 47.0 (–*C*H(CH_3_)–S–), 41.8 (–*C*–CH_2_–), 39.0 (–S*C*H_2_–), 21.4 (–*C*H_2_CH_3_), 16.3 (–CH*C*H_3_), 13.1 (–CH_2_*C*H_3_). Elemental analysis: C 40.67 (cal. 41.25), H 5.67 (cal. 5.42), S 39.37 (cal. 40.00).

### 2.4. RAFT Polymerization of NIPA

Tetra-arm PNIPAs (T-PNIPAs) with four trithiocarbonate terminal groups were prepared based on RAFT polymerization of NIPA in the presence of star-CTA. Typically, NIPA (2.26 g, 20 mmol), star-CTA (114 mg, 0.12 mmol) and AIBN (3.9 mg, 0.024 mmol) were dissolved in 6.8 g of distilled THF in a 30 mL Schlenk tube. The mixture was subjected to three freeze-pump-thaw cycles and was stirred under nitrogen atmosphere in 70 °C water bath. The polymerization progress was monitored by extracting 0.5 mL of aliquots from the reaction solution at the desired time interval. The extracted solutions were immediately quenched by opening in air and cooling in liquid nitrogen. The solvent was removed under a gentle airstream and subsequent vacuum drying. The resulting solid was used for ^1^H NMR measurement. The monomer conversion was calculated from the areas of signals at 5.6 ppm due to the vinyl proton of NIPA and the areas sum of the isopropyl methine proton signals at 4.0 and 4.1 ppm, corresponding to PNIPA and the residual NIPA monomer, respectively.

PNIPAs with different molecular weights were prepared through changing the dosage of compounds and polymerization time—the detail was listed in Table 1. At the end of polymerization, the resultant was quickly quenched by opening in air and cooling in liquid nitrogen. Then, 0.5 mL of the resultant was taken out for the determination of monomer conversion, and the left was rotary-evaporated to concentrate, followed by precipitation in diethyl ether. The obtained yellow solid was purified further by two consecutive reprecipitations from THF into diethyl ether.

### 2.5. Preparation of Tetra-propargyl Terminal PNIPAs (TP-PNIPAs)

The trithiocarbonate groups at the end of tetra-arm PNIPAs were modified to propargyl moieties following the method described in the literature [19]. Typically, 0.96 g of yellow T-PNIPA16K (0.24 mmol trithiocarbonate groups) and 5 mL of THF were charged into a 30 mL Schlenk tube, which was degassed and filled with nitrogen through three freeze-pump-thaw cycles. Then, a solution of *n*-butylamine (90 mg, 1.2 mmol) in 0.2 g of THF, which was purged with nitrogen in advance, was charged into polymer solution, followed by the addition of TCEP–HCl (5 mg). The mixture was stirred for 1 h at room temperature under a nitrogen atmosphere. Subsequently, a solution containing PA (264 mg, 2.4 mmol) and THF (0.25 g) was added, and the mixture was stirred under nitrogen protection for 24 h at room temperature. Finally, the polymer TP-PNIPA16K was recovered and purified by reprecipitation from THF into diethyl ether three times, and 0.9 g of white solids was obtained, yield = 94%. Similarly, TP-PNIPA7K and TP-PNIPA24K were prepared, with yield of 89% and 93%, respectively.

### 2.6. Estimation of PNIPA Molecular Weights

The molecular weights (*M*_n_) of RAFT T-PNIPA samples were obtained by the methods as described in our previous paper [25]. Equation (1) was used to calculate *M*_n_(theory) based on monomer conversion that was estimated according to ^1^H NMR spectra of unseparated polymerizing resultant including polymer and residual monomers. For the purified polymers, *M*_n_(NMR) was evaluated via Equation (2) using the peak areas at 3.3 and 4.0 ppm, which correspond to propyl methylene adjacent to trithiocarbonate and isopropyl methine protons, respectively. *M*_n_(UV–Vis) was derived as follows: accurately weighed T-PNIPA sample (~20 mg) was dissolved in 25 mL of DCM to obtain solution with a known polymer concentration. The UV absorbance of the solution at 310 nm was measured. In addition, the concentration of the trithiocarbonate group was calculated according to the linear relationship of absorbance (at 310 nm) and star-CTA concentration in DCM, which was derived as Equation (3). Further, *M*_n_ was reckoned applying Equation (4).

(1) *M*_n_(theory) = conversion × ([NIPA]/[CTA]) × *M*_NIPA_ + *M*_CTA_

(2) *M*_n_(NMR) = 8 × (*I*_4.0ppm_/*I*_3.3ppm_) × *M*_NIPA_ + *M*_CTA_

(3) *C*_CTA_ = 2.65 × 10^−5^*A*

(4) *M*_n_(UV–Vis) = *ρ*/*C*_CTA_

where *M*_NIPA_ and *M*_CTA_ mean individual molecular weights of NIPA and CTA, whose values are 113 and 960, respectively; *I*_4.0ppm_ and *I*_3.3ppm_ refer to peak areas at 4.0 and 3.3 ppm in obtained PNIPA ^1^H NMR spectra, respectively, which belong to isopropyl methine and *n*-propyl methylene protons; *C*_CTA_ stands for CTA concentration in DCM (mol/L), *A* is absorbance at 310 nm recorded by UV–Vis, and *ρ* represents polymer concentration in DCM (g/L).

For TP-PNIPA, whose terminal trithiocarbonate groups have been converted to propargyls after modification, *M*_n_ was calculated following Equation (5) based on ^1^H NMR spectrum.

(5) *M*_n_(NMR) = 8 × (*I*_4.0ppm_/*I*_4.7ppm_) × *M*_NIPA_ + *M*_CTA_

where *I*_4.0ppm_ and *I*_4.7ppm_ refer to peak areas at 4.0 and 4.7 ppm, respectively, which correspond to isopropyl methine and propargyl methylene protons.

### 2.7. Temperature Sensitivities of PNIPAs in Aqueous Solution

Towards prepared PNIPA samples, aqueous solutions with concentration of 0.75 mg/mL were made. They were used to study temperature sensitivities applying a UV–Vis spectrometer equipped with a temperature controller system. The UV–Vis transmittance at 500 nm for a polymer solution in a 1 cm path length quartz cell was recorded during temperature changing from 20 to 40 °C at a heating rate of 0.2 °C/min; meanwhile, the solution temperature was monitored with an internal probe.

### 2.8. Fabrication of PNIPA Hydrogels

In a flat-bottom screw-capped glass vial (ϕ 1 cm), the predetermined amount of TP-PNIPA and β-CD-(N_3_)_7_, total weight of which was 200 mg, were dissolved in 750 mg of DMF. After uniform mixing, 25 μL of 0.5 M CuSO_4_ aqueous solution and 25 μL of 1.0 M ascorbic acid in DMF were added in turn. Then, the vial was sealed and shaken, and the gel formed within 15–30 min, which was left for 24 h. Afterwards, the gel was taken out to immerse in DMF for 5 day and in 0.2 M ethylene diamine tetraacetic acid disodium salt (EDTA·2Na) aqueous solution for another 5 d in order to remove unreacted precursors and copper ions, respectively. Subsequently, the gel was swollen in deionized water for 5 day before being dried and weighed. Here, the soaking medium was exchanged daily during immersion. The mass of xerogel was divided by 200 to get gel fraction. Through changing the species of TP-PNIPAs, several hydrogels were prepared.

### 2.9. Swelling Properties of Hydrogels

Pre-weighted xerogels were immersed in deionized water at 10 °C, and some time later, they were taken out and weighed after removal of excess surface water with wet filter paper. The equilibrium was reached until weight of a swollen gel kept constant. The equilibrium swelling ratio (*ESR*) value was calculated with Equation (6).

(6) *ESR* = (*m*_e_ − *m*_0_)/*m*_0_

where *m*_0_ and *m*_e_ are the weights of xerogel and equilibrated swollen hydrogel, respectively.

Afterwards, the temperature was increased step by step to 40 °C by changing the bath temperature. The gels were kept at a certain temperature for 48 h to ensure swelling equilibrium, and *ESR* values at every temperature point were obtained. In all of the swelling ratio measurements, three hydrogel samples of the same formulation were used, and the average value was taken.

### 2.10. Characterization

^1^H NMR spectra were obtained using Bruker DRX-500 (Bruker, Rheinstetten, Germany) with CDCl_3_ or DMSO-*d*_6_ as solvent. GPC measurements were carried out at room temperature on a Waters instrument (Waters, Milford, CT, USA) attached to a 2414 refractive index detector, using DMF as eluent at a flow rate of 1.0 mL/min, with polystyrene as standards. Elemental analysis of C, H, N and S was performed on an Elementar vario EL III (Elementar, Hanau, Germany). A UV–Vis absorption spectroscopy was performed on PerkinElmer UV–Vis spectrometer (Lambda 35) (PerkinElmer, Waltham, MA, USA). The morphology of hydrogel networks was characterized by scanning electron microscopy (SEM, JSM-6700F) (JEOL, Peabody, MA, USA). The swollen hydrogels at 10 °C were lyophilized, and sputtered with gold before SEM observation.

## 3. Results and Discussion

### 3.1. Preparation of Telechelic Tetra-Arm PNIPAs through RAFT Polymerization

**Figure 1:**
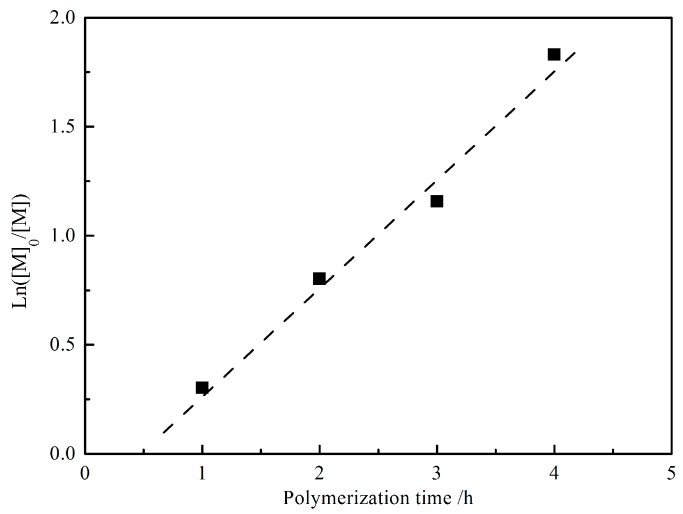
Pseudo-first-order kinetic plot of PNIPA RAFT polymerization. The polymerization condition is as follows: [NIPA]/[star-CTA]/[AIBN] = 167/1/0.2, the monomer concentration in THF is 25 wt %, polymerization temperature is 70 °C.

In order to obtain a series of well-defined tetra-arm PNIPAs with different molecular weights via RAFT polymerization, a tetra-arm star-CTA containing a trithiocarbonate moiety at every end was synthesized according to the method described in the literature [12,28]. This compound was prepared through two steps with PTB as an intermediate, and their structures were confirmed by ^1^H and ^13^C NMR, as shown in Figures S3 and S4 (Supplementary Material). In the presence of the star-CTA, RAFT polymerization of NIPA was performed in THF at 70 °C with AIBN as initiator. Figure 1 displays the polymerization kinetics for a certain formulation, in accordance with the basic characteristics of living polymerization. Here, ln([*M*]_0_/[*M*]) was derived from monomer conversion that was calculated on the basis of relevant peak areas revealed in Figure S5 (Supplementary Material), via the method described in Section 2.4.

**Figure 2:**
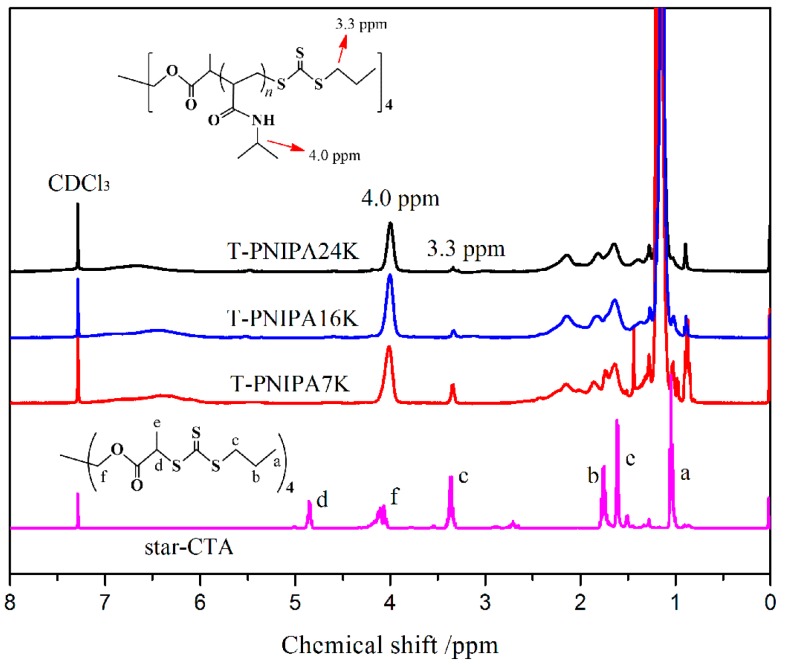
^1^H NMR spectra of star-CTA and T-PNIPAs with terminal trithiocarbonate groups.

RAFT polymerization parameters were altered, preparing three well-defined tetra-arm PNIPAs with different molecular weights. Figure 2 shows ^1^H NMR spectra of purified T-PNIPAs together with star-CTA, confirming the existence of corresponding groups of CTA in polymers. In addition, molecular weights were estimated using relevant peak areas. The detailed polymerization conditions and some characterizations of prepared polymers are listed in Table 1. One can see that the molecular weights calculated by various methods show close values, indicating the well-defined nature of obtained PNIPAs in combination with the measured PDI values lower than 1.20. Molecular weights obtained by GPC are not listed here because the values are relative to the standards, deviating from real ones more greatly. These polymers were nominated as T-PNIPA7K, 16K and 24K, respectively, based on their molecular weights.

### 3.2. Modification of RAFT PNIPAs

**Figure 3:**
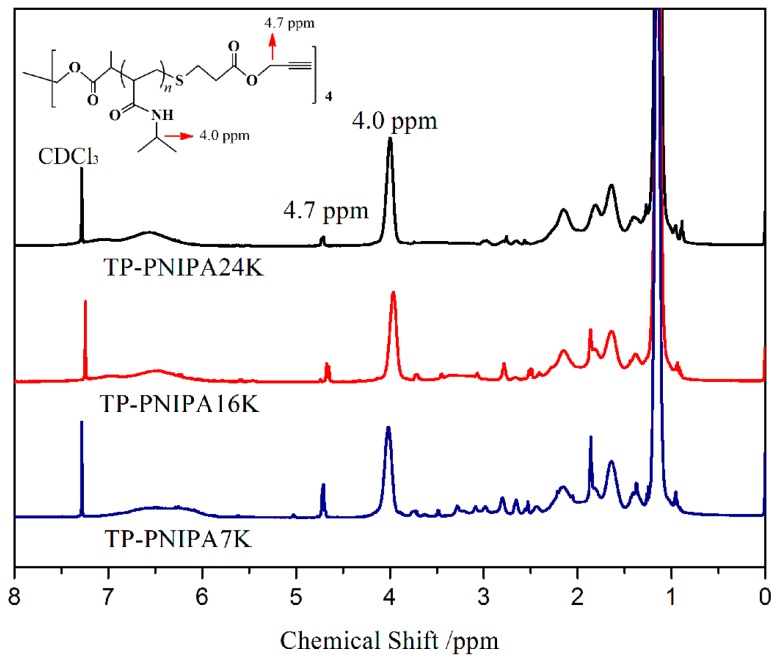
^1^H NMR spectra of TP-PNIPAs with terminal propargyl groups.

Terminal trithiocarbonate groups on well-defined RAFT T-PNIPA chains provide the chance of further functionalization. As mentioned in the Introduction section, aminolysis/Michael addition treatment is a usual approach to realize thiocarbonate transformation. In this study, T-PNIPAs were subjected to aminolysis of *n*-butylamine and, following Michael addition, with propargyl acrylate under the catalysis of TCEP–HCl, referring to the procedure described by Qiu and co-workers [19]. Finally, tetra-arm star PNIPAs with propargyl end groups, TP-PNIPAs, were achieved, and their ^1^H NMR spectra are shown in Figure 3. According to the method described in Section 2.6, their molecular weights were estimated, revealed in Table 2. The close values of molecular weights for TP-PNIPAs and their precursors, together with UV and GPC trace comparison before and after modification (Figures S6 and S7, Supplementary Material), reflects successful transformation without undesired side reactions.

### 3.3. Temperature Sensitivities of Tetra-Arm PNIPAs in Aqueous Solution

**Figure 4:**
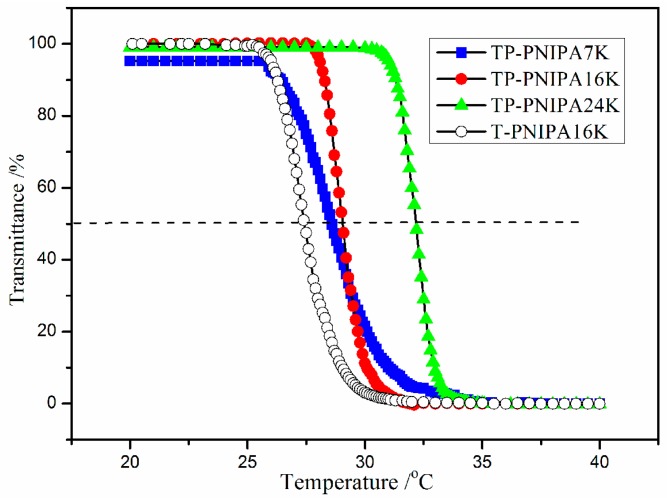
Solution transparency at 500 nm as a function of temperature of PNIPA solutions (0.75 mg/mL).

Solution transmittance alternation with temperature of PNIPAs was explored, as plotted in Figure 4. According to the analysis of PNIPA solutions reported by others [21,29,30,31], temperature sensitive properties of obtained polymers in this work are studied with respect to the starting and ending temperature points (*T*_LCST,min_, *T*_LCST,max_) of phase transition and cloud point (*T*_CP_). Here, both *T*_LCST,min_ and *T*_LCST,max_ values are extracted from two inflection points of transmittance-temperature curves, respectively; *T*_CP_ is defined as the temperature corresponding to 50 % decrease of transmittance. The respective values of three temperature points are listed in Table 2. It can be observed that *T*_LCST,min_ and *T*_CP_ values of TP-PNIPA samples increase with their molecular weights, while *T*_LCST,max_ values are comparable.

With regard to the changing trend of phase transition temperatures (PTT) such as LCST or CP with polymer chain lengths for aqueous PNIPA solution, different results have been reported. For example, originally Fujishige *et al.* concluded that PNIPA molecular weight has no influence on LCST [32]. However, the inverse effect of molecular weight on LCST was found for PNIPAs with various structures including linear, cyclic and star ones [30,33,34,35]. This property is reasonably ascribed to the distinction in polymer-water interactions: longer chain means greater molar volumes of polymer in solution, which will reduce the critical value of the Flory–Huggins interaction parameter that is known to be proportional to LCST [36]. In addition, the contrary phenomenon, *i.e.*, LCST becomes higher with PNIPA chain lengths, was also reported [21,37,38,39]. Such a property is in agreement with that of PNIPAs studied in this work.

Actually, the effect of chain length on LCST relies on two factors such as polymer-water interaction and the hydrophobic/hydrophilic balance of the polymer, as elucidated by others [21,35]. It is well-known that the incorporation of hydrophobic and hydrophilic components to PNIPAs will induce the decrease and increase of LCST, respectively. The reason is that the introduced hydrophobic segments can improve the dehydration of isopropyls and accompanied hydrophobic interactions with raising temperature, and *vice versa*. Herein, for the obtained TP-PNIPAs, the hydrophobic parts contain adducts of mercaptan with propargyl acrylate at four ends, as well as ester moieties surrounding the core. The lower molecular weight of polymer implies the greater relative content of the two hydrophobic components above, which, in turn, depresses LCST value. For instance, *T*_LCST,min_ values are lowered from 30 to 25.5 °C when TP-PNIPA molecular weights are reduced from 24 to 7 K; TP-PNIPA24K has a *T*_CP_ value of 32 °C, a commonly reported value for conventional PNIPA [35], while *T*_CP_ decreases to around 29 °C for both TP-PNIPA16K and TP-PNIPA7K. To further validate the effect of hydrophobicity on PTT, TP-PNIPA16 was compared with its precursor T-PNIPA16. Without the effect of molar mass, the only difference is that the latter’s terminal trithiocarbonate groups were modified into propargyl ester moieties to get the former one, so T-PNIPA16 is more hydrophobic than TP-PNIPA16. When T-PNIPA16 was transformed into TP-PNIPA16, *T*_LCST,min_ and *T*_CP_ are shifted upward by 2.6 and 1.5 °C, respectively, as shown in Figure 4 and Table 2.

Another important piece of information that needs to be mentioned is transition breadth, *i.e.*, Δ*T*, a deviation of *T*_LCST,min_ and *T*_LCST,max_, which may be related with aggregation rate of PNIPA chains [34]. From Table 2, one can find that Δ*T* appears to have a similar trend to that of *T*_LCST,min_ or *T*_CP_. This should also arise from their difference in relative hydrophobicities. The same characteristics were also reported in Xia and colleagues’ work about narrow-disperse PNIPAs, especially for low-molar-mass ones, in which the influence of terminal hydrophopobic groups becomes significant [34,35].

### 3.4. Fabrication and Temperature Sensitivities of Click PNIPA Hydrogels

A series of PNIPA hydrogels were constructed via CuAAC utilizing β-CD-(N_3_)_7_ to crosslink well-defined TP-PNIPAs with terminal propargyls. Corresponding to molecular weights, the obtained hydrogels were named as G7K, G16K and G24K, respectively. In hydrogel formulation, two azido groups per β-CD molecule were designed to participate in click reaction, aiming to get well-defined regular networks. That is to say, two PNIPA chains from different macromolecule units were planned to interlink through one modified β-CD molecule as a knot (Scheme 1). Of course, this supposition is only an ideal state because there are seven azido groups on a CD molecule, after all. However, this ideal hypothesis is of probable existence due to the low concentration of raw materials (20 wt %); and we have tried to lower the concentration to 15 wt %, finding that the obtained gel almost cannot self-stand. Herein, the gelation was accomplished in half an hour using 20 wt % polymer solution in DMF, and the final gel fraction of each sample was over 90 %, under the catalysis of a common CuSO_4_/ascorbic acid system, the same as our previously published works [24,25]. In a study about click PNIPA hydrogels [21], however, towards PNIPA solutions in DMF with as high as 80 wt % concentration, the authors finally selected CuI/*N*,*N*-diisopropylethylamine (DIPEA) pair through examining many catalyst systems including CuSO_4_ and Cu(I) salts in combination with various reducing agents and ligands because only CuI/DIPEA system behaved highly efficient. They ascribed this abnormal founding to the chelation of acrylamide groups to copper ions [21], but we think a more important reason might be the influence of molecular structures, especially for the microenvironment near acetylene and azide moieties. After all, most CuAAC PNIPA hydrogels had successfully adopted CuSO_4_/ascorbic acid system [23,40,41].

**Figure 5:**
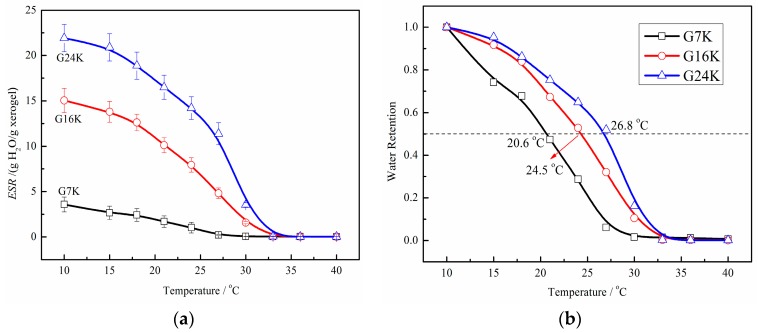
(**a**) The effect of temperature on *ESR* of click PNIPA hydrogels; (**b**) water retention of samples at different temperatures relative to *ESR* at 10 °C.

The swelling properties of click PNIPA hydrogels in terms of temperature dependence were researched, and the *ESR vs.* temperature curves are plotted in Figure 5a. As expected, hydrogels made from higher molecular-weight PNIPAs have greater *ESR* values in swollen state. As have been elaborated in our previous paper [25], the higher molecular weight of PNIPA implies longer distance between crosslinks, which will provide larger free volume to accommodate more water molecules. On the other hand, the higher the PNIPA molecular weight is, the less is the hydrophobic parts content; meanwhile the dosage of less hydrophilic azido-β-CD crosslinker also decreases in gel fabrication. It is these combined actions that induce such a trend of swelling ratios with molecular weights of used PNIPAs.

As expected, the obtained PNIPA hydrogels displayed obvious deswelling behaviors with increasing temperature, shown in Figure 5a. Correspondingly, the appearances of gels also altered from transparent expansive states to white huddled ones gradually at elevated temperatures; that is to say, the inherent homogeneous state of swollen gels became heterogeneous ascribed to the aggregation of PNIPA chains in network as the temperature rose. Volume phase transition temperature (VPTT), a parameter to characterize temperature sensitive hydrogels, can be regarded as the temperature at which the swelling ratio decreased to a half of its value at initial temperature [25]. Therefore, the curves of *ESR vs.* temperature in Figure 5a were normalized based on the data at 10 °C, as displayed in Figure 5b, where water retention was defined as the ratio between *ESR* at a certain temperature and that at 10 °C. From Figure 5b, VPTT values of G7K, G16K and G24K are evaluated to be 20.6, 24.5 and 26.8 °C, respectively. This result appears identical to the trend of *T*_LCST,min_ or *T*_CP_ for aqueous TP-PNIPA solutions discussed above. Undoubtedly, the hydrophobic/ hydrophilic balance within gel networks takes crucial responsibility for the tendency of VPTT with molecular weights of PNIPAs used for gelation. Such a feature of VPTT and *ESR* values’ variation with PNIPA molecular weights also supplies a facile approach to regulate swelling and responsive properties of PNIPA-based hydrogels for different applications.

### 3.5. SEM Photographs of Freeze-Dried Hydrogel Networks

**Figure 6:**
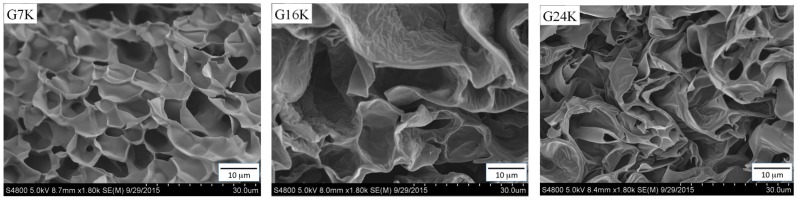
SEM photographs of freeze-dried gel samples.

In order to observe internal network structures of obtained hydrogels, the swollen samples at 10 °C were freeze-dried, followed by SEM characterization, as shown in Figure 6. It can be seen that G7K appears to have a regular porous structure with small size, while the pore size of G16K becomes bigger but more irregular, and that of G24K seems somewhat more compact and less uniform than the former two. It has been known that hydrogels made from TP-PNIPAs with greater molecular weights have higher *ESR* values at swollen state; thus, they are in favor of forming greater ice crystals within networks during freezing. In this consideration, higher swelling ratios for hydrogels should correspond to bigger pores inside freeze-dried networks. However, simultaneously, higher water content also causes thinner pore walls because the polymer fraction in hydrogels becomes lower, which, in turn, probably leads to weaker supporting forces of networks; subsequently, the partial shrinking and/or collapse may happen during the course of freeze-drying [25,42]. Thus, the appeared SEM images of three samples become easy to understand.

## 4. Conclusions

A series of well-defined tetra-arm telechelic PNIPAs with propargyl terminal groups were synthesized via RAFT polymerization and further modification. Their structures were confirmed by relevant characterizations. The temperature sensitivities of obtained tetra-arm PNIPAs were investigated through turbidity analysis of their aqueous solutions. For TP-PNIPAs with the same terminal moieties, the increment of molecular weights renders PTTs (*T*_LCST,min_ and *T*_CP_) to shift upward to higher temperatures; TP-PNIPA16K also shows higher PTTs than its precursor T-PNIPA16K because the former is less hydrophobic than the latter; in addition, the transition breadths of samples are also dependent on their structures. The results demonstrate that the hydrophobic/hydrophilic balance determines the temperature sensitive behaviors of tetra-arm PNIPAs studied in this presentation.

Furthermore, three PNIPA hydrogels were fabricated through click chemistry of obtained TP-PNIPAs with β-CD-(N_3_)_7_ utilizing the common CuSO_4_/ascorbic acid system. Their temperature sensitivities display similar characteristics to those of TP-PNIPAs, *i.e.*, both swelling ratios and VPTTs are closely connected with the relative hydrophilicity or molecular weights of TP-PNIPAs. Correspondingly, this study provides a facile method to modulate swelling and responsive behaviors of smart PNIPA hydrogels. It is also necessary to mention that β-CD is another component besides the major PNIPA within gel networks, the incorporation of which can endow the hydrogels with additional or improved properties due to inherent inclusion and adsorption of CD molecules, and this feature is rather favorable for designing intelligent materials in applications of separation and carrier. In addition, residual azide groups besides those for crosslinking also supply a platform for introducing other functional groups simultaneously, as we have done in our previous study [25]. The relevant work is going to be done in future.

## Figures and Tables

**Table 1:** The detailed information of obtained T-PNIPAs.

Samples	[NIPA]/[Star- CTA]/[AIBN]	Polymerization time (min)	Conversion (%)	*M*_n_ (kg/mol)			PDI (GPC)
				Theory ^*a*^	^1^H NMR ^*b*^	UV–Vis ^*c*^	
T-PNIPA7K	80/1/0.2	120	68	7.1	6.7	6.8	1.14
T-PNIPA16K	167/1/0.2	240	84	16.8	16.3	17.3	1.12
T-PNIPA24K	250/1/0.2	270	82	24.1	25.4	23.5	1.18

^*a*^ Calculated according to Equation (1); ^*b*^ Derived based on Equation (2); ^*c*^ Reckoned with Equation (4).

**Table 2:** Some properties of TP-PNIPAs and T-PNIPA16K.

Samples	*M*_n_^a^ (kg/mol)	PDI	*T*_LCST,min_ (°C)	*T*_LCST,max_ (°C)	Δ*T* (°C)	*T*_CP_^b^ (°C)
TP-PNIPA7K	7.4	1.16	25.5	33.0	7.5	28.5
TP-PNIPA16K	16.5	1.18	27.8	32.0	4.2	29.0
TP-PNIPA24K	25.0	1.20	30.0	33.5	3.5	32.0
T-PNIPA16K	16.3	1.12	25.2	31.0	5.8	27.5

^*a*^ Estimated according to Equation (5) for TP-PNIPAs and Equation (2) for T-PNIPA16K; ^b^ Temperature at which transmittance decreases to 50 %.

## Supplementary Materials

Supplementary Materials can be found at www.mdpi.com/2073-4360/8/4/93/s1.

## References

[1] Matyjaszewski K. (2012). Atom transfer radical polymerization (ATRP): Current status and future perspectives. Macromolecules.

[2] Nicolas J., Guillaneuf Y., Lefay C., Bertin D., Gigmes D., Charleux B. (2013). Nitroxide-mediated polymerization. Prog. Polym. Sci..

[3] Moad G., Rizzardo E., Thang S.H. (2013). RAFT polymerization and some of its applications. Chem. Asian J..

[4] Harvison M.A., Lowe A.B. (2011). Combining RAFT radical polymerization and click/highly efficient coupling chemistries: A powerful strategy for the preparation of novel materials. Macromol. Rapid Commun..

[5] Roth P.J., Boyer C., Lowe A.B., Davis T.P. (2011). RAFT polymerization and thiol chemistry: A complementary pairing for implementing modern macromolecular design. Macromol. Rapid Commun..

[6] Moad G., Rizzardo E., Thang S.H. (2011). End-functional polymers, thiocarbonylthio group removal/transformation and reversible addition-fragmentation-chain transfer (RAFT) polymerization. Polym. Int..

[7] Bassik N., Abebe B.T., Laflin K.E., Gracias D.H. (2010). Photolithographically patterned smart hydrogel based bilayer actuators. Polymer.

[8] Gu Y.D., Dhanarajan A.P., Hruby S.L., Baldi A., Ziaie B., Siegel R.A. (2009). An interpenetrating glass-thermosensitive hydrogel construct gated flow control and thermofluidic oscillations. Sens. Actuators B Chem..

[9] Deshmukh M.V., Vaidya A.A., Kulkarni M.G., Rajamohanan P.R., Ganapathy S. (2000). LCST in poly(*N*-isopropylacrylamide) copolymers: High resolution proton NMR investigations. Polymer.

[10] Liu B., Wang H., Zhang L., Yang G., Liu X., Kim I. (2013). A facile approach for the synthesis of cyclic poly(*N*-isopropylacrylamide) based on an anthracene–thiol click reaction. Polym. Chem..

[11] Qiu X.-P., Korchagina E.V., Rolland J., Winnik F.M. (2014). Synthesis of a poly(*N*-isopropylacrylamide) charm bracelet decorated with a photomobile α-cyclodextrin charm. Polym. Chem..

[12] Tao L., Kaddis C.S., Loo R.R.O., Grover G.N., Loo J.A., Maynard H.D. (2009). Synthesis of maleimide-end-functionalized star polymers and multimeric protein–polymer conjugates. Macromolecules.

[13] Wei Z., Zhu S.Z., Zhao H.Y. (2015). Brush macromolecules with thermo-sensitive coil backbones and pendant polypeptide side chains: Synthesis, self-assembly and functionalization. Polym. Chem..

[14] Tao W., Yan L.F. (2010). Thermogelling of highly branched poly(*N*-isopropylacrylamide). J. Appl. Polym. Sci..

[15] Lv W., Liu S., Feng W., Qi J., Zhang G., Zhang F., Fan X. (2011). Temperature- and redox-directed multiple self assembly of poly(*N*-isopropylacrylamide) grafted dextran nanogels. Macromol. Rapid Commun..

[16] Zhu M.Q., Wang L.Q., Exarhos G.J., Li A.D.Q. (2004). Thermosensitive gold nanoparticles. J. Am. Chem. Soc..

[17] Huang A., Qin G.K., Olsen B.D. (2015). Highly active biocatalytic coatings from protein-polymer diblock copolymers. ACS Appl. Mater. Interfaces.

[18] Shi G.Y., Tang X.Z., Pan C.Y. (2008). Tadpole-shaped amphiphilic copolymers prepared via RAFT polymerization and click reaction. J. Polym. Sci. Part A.

[19] Qiu X.-P., Winnik F.M. (2006). Facile and efficient one-pot transformation of RAFT polymer end groups via a mild aminolysis/michael addition sequence. Macromol. Rapid Commun..

[20] Yu B., Chan J.W., Hoyle C.E., Lowe A.B. (2009). Sequential thiol-ene/thiol-ene and thiol-ene/thiol-yne reactions as a route to well-defined mono and bis end-functionalized poly(*N*-isopropylacrylamide). J. Polym. Sci. Part A.

[21] Ooi H.W., Jack K.S., Peng H., Whittaker A.K. (2013). “Click” PNIPAAm hydrogels—A comprehensive study of structure and properties. Polym. Chem..

[22] Ooi H.W., Jack K.S., Whittaker A.K., Peng H. (2013). Photo-initiated thiol-ene “click” hydrogels from RAFT-synthesized poly(*N*-isopropylacrylamide). J. Polym. Sci. Part A.

[23] Pasale S.K., Cerroni B., Ghugare S.V., Paradossi G. (2014). Multiresponsive hyaluronan-P(NIPAAm) “click”-linked hydrogels. Macromol. Biosci..

[24] Wang J., Zhang Z., Liu Y., Lv Y., Shao Z. (2015). Poly(*N*-isopropylacrylamide) hydrogels crosslinked by small-molecular crosslinkers through click chemistry. Int. J. Polym. Mater. Polym. Biomater..

[25] Wang J.Q., Kang Z.Y., Qi B., Zhou Q.S., Xiao S.Y., Shao Z.Q. (2014). Poly(*N*-isopropylacrylamide) hydrogels fabricated via click chemistry: Well-defined α,ω-bis propargyl linear poly(*N*-isopropylacrylamide)s as crosslinkers. RSC Adv..

[26] Le H.T., Jeon H.M., Lim C.W., Kim T.W. (2014). 6-Triazolyl-6-deoxy-β-cyclodextrin derivatives: Synthesis, cellular toxicity, and phase-solubility study. Carbohydr. Res..

[27] Becker M.M., Ravoo B.J. (2010). Highly fluorinated cyclodextrins and their host-guest interactions. Chem. Commun..

[28] Zayas H.A., Truong N.P., Valade D., Jia Z., Monteiro M.J. (2013). Narrow molecular weight and particle size distributions of polystyrene 4-arm stars synthesized by RAFT-mediated miniemulsions. Polym. Chem..

[29] Xu J., Ye J., Liu S.Y. (2007). Synthesis of well-defined cyclic poly(*N*-isopropylacrylamide) via click chemistry and its unique thermal phase transition behavior. Macromolecules.

[30] Qiu X.P., Tanaka F., Winnik F.M. (2007). Temperature-induced phase transition of well-defined cyclic poly(*N*-isopropylacrylamide)s in aqueous solution. Macromolecules.

[31] Jain K., Vedarajan R., Watanabe M., Ishikiriyama M., Matsumi N. (2015). Tunable LCST behavior of poly(*N*-isopropylacrylamide/ionic liquid) copolymers. Polym. Chem..

[32] Fujishige S., Kubota K., Ando I. (1989). Phase-transition of aqueous-solutions of poly(*N*-isopropylacrylamide) and poly(*N*-isopropylmethacrylamide). J. Phys. Chem. Us.

[33] Chen Y.G., Xiao N., Fukuoka M., Yoshida K., Duan Q., Satoh T., Kakuchi T. (2015). Synthesis and thermoresponsive properties of four-arm star-shaped poly(*N*-isopropylacrylamide)s bearing covalent and non-covalent cores. Polym. Chem..

[34] Xia Y., Burke N.A.D., Stover H.D.H. (2006). End group effect on the thermal response of narrow-disperse poly(*N*-isopropylacrylamide) prepared by atom transfer radical polymerization. Macromolecules.

[35] Xia Y., Yin X.C., Burke N.A.D., Stover H.D.H. (2005). Thermal response of narrow-disperse poly(*N*-isopropylacrylamide) prepared by atom transfer radical polymerization. Macromolecules.

[36] Lessard D.G., Ousalem M., Zhu X.X. (2001). Effect of the molecular weight on the lower critical solution temperature of poly(*N*,*N*-diethylacrylamide) in aqueous solutions. Can. J. Chem..

[37] Zheng X., Tong Z., Xie X.L., Zeng F. (1998). Phase separation in poly(*N*-isopropyl acrylamide) water solutions I. Cloud point curves and microgelation. Polym. J..

[38] Plummer R., Hill D.J.T., Whittaker A.K. (2006). Solution properties of star and linear poly(*N*-isopropylacrylamide). Macromolecules.

[39] Kujawa P., Segui F., Shaban S., Diab C., Okada Y., Tanaka F., Winnik F.M. (2006). Impact of end-group association and main-chain hydration on the thermosensitive properties of hydrophobically modified telechelic poly(*N*-isopropylacrylamides) in water. Macromolecules.

[40] Xu X.D., Chen C.S., Wang Z.C., Wang G.R., Cheng S.X., Zhang X.Z., Zhuo R.X. (2008). “Click” chemistry for *in situ* formation of thermoresponsive P(NIPAAm-*co*-HEMA)-based hydrogels. J. Polym. Sci. Part A.

[41] Xu X.D., Chen C.S., Lu B., Wang Z.C., Cheng S.X., Zhang X.Z., Zhuo R.X. (2009). Modular synthesis of thermosensitive P(NIPAAm-*co*-HEMA)/β-CD based hydrogels via click chemistry. Macromol. Rapid Commun..

[42] Zhang N., Shen Y., Li X., Cai S., Liu M. (2012). Synthesis and characterization of thermo- and pH-sensitive poly(vinyl alcohol)/poly(*N*,*N*-diethylacrylamide-*co*-itaconic acid) semi-IPN hydrogels. Biomed. Mater..

