# Supplementary material for: Preparation of Well-Defined Propargyl-Terminated Tetra-Arm Poly(N-isopropylacrylamide)s and Their Click Hydrogels Crosslinked with β-cyclodextrin
Source: Polymers (Basel). 2016 Mar 28;8(4):93. doi: 10.3390/polym8040093 (PMC6432514; doi:10.3390/polym8040093)
Supplement: Supplementary file 1 [file polymers-08-00093-s001.pdf]

Supplementary Materials: Preparation of Well-defined Propargyl-terminated Tetra-arm Poly(*N*-isopropylacrylamide)s and Their Click Hydrogels Crosslinked with β-cyclodextrin

Jianquan Wang, Zhe Zhu, Xin Jin, Zhujun Li, Yizhen Shao and Ziqiang Shao

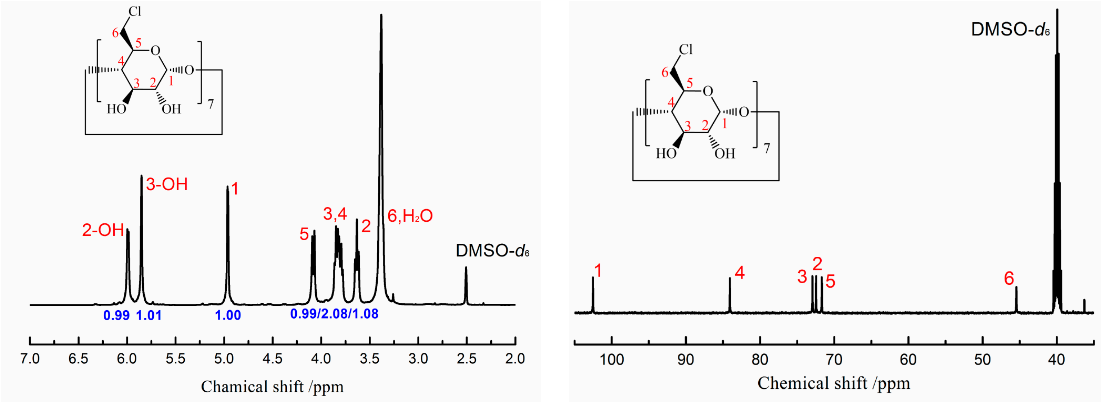

(**a**) (**b**)

Figure S1. ^{1}H NMR (**a**) and ^{13}C NMR (**b**) spectra of β-CD-Cl$_7$.

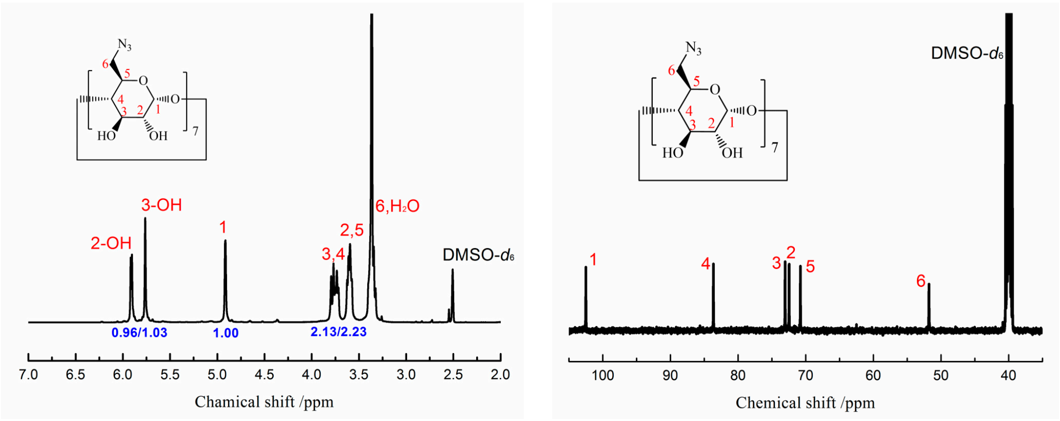

(**a**) (**b**)

Figure S2. ^{1}H NMR (**a**) and ^{13}C NMR (**b**) spectra of β-CD-(N$_3$)$_7$.

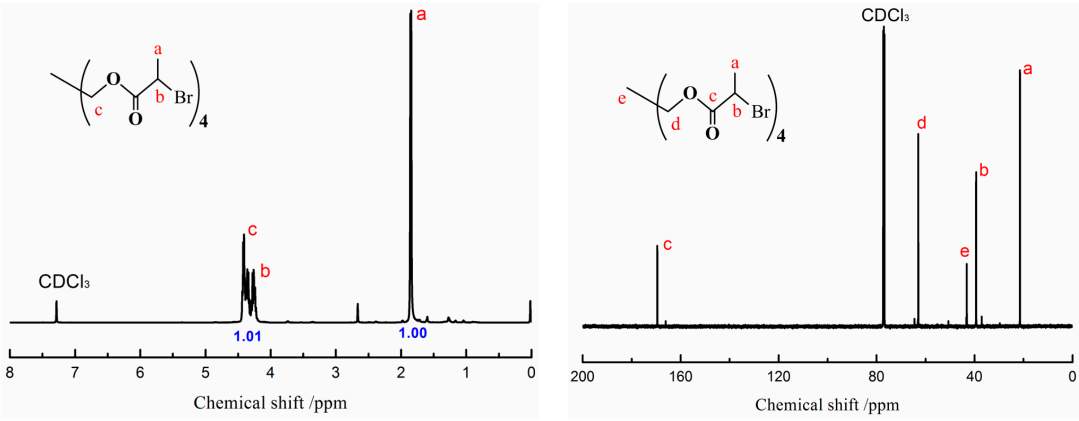

(**a**) (**b**)

Figure S3. ^{1}H NMR (**a**) and ^{13}C NMR (**b**) spectra of PTB.

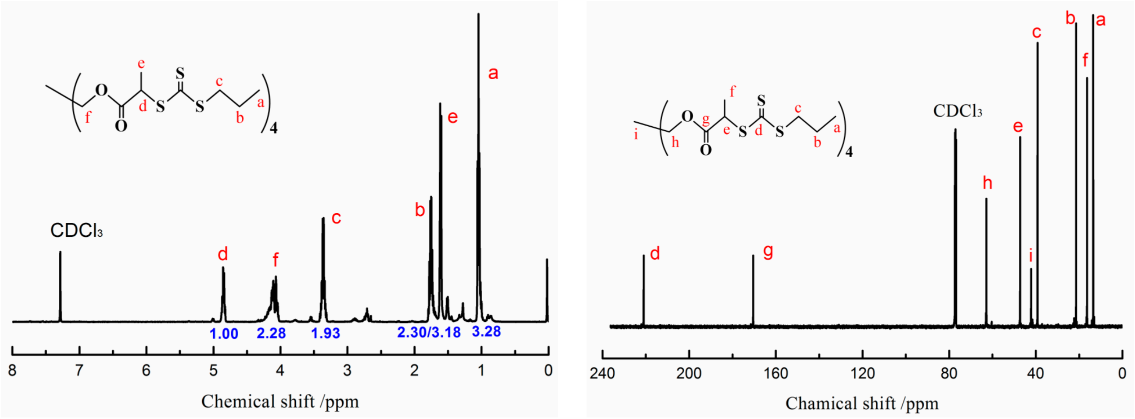

(**a**) (**b**)

Figure S4. ^{1}H NMR (**a**) and ^{13}C NMR (**b**) spectra of star-CTA.

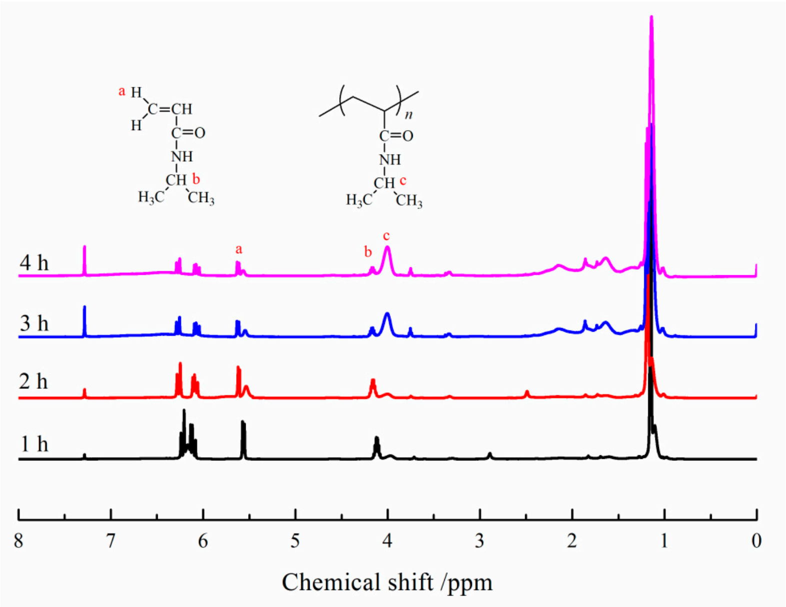

Figure S5. ^{1}H NMR spectra of NIPA polymerization systems at different times, which was used to calculate monomer conversion. The polymerization condition is as follows: [NIPA]/[star-CTA]/[AIBN]=167/1/0.2, the monomer concentration in THF is 25 wt %, polymerization temperature is 70°C.

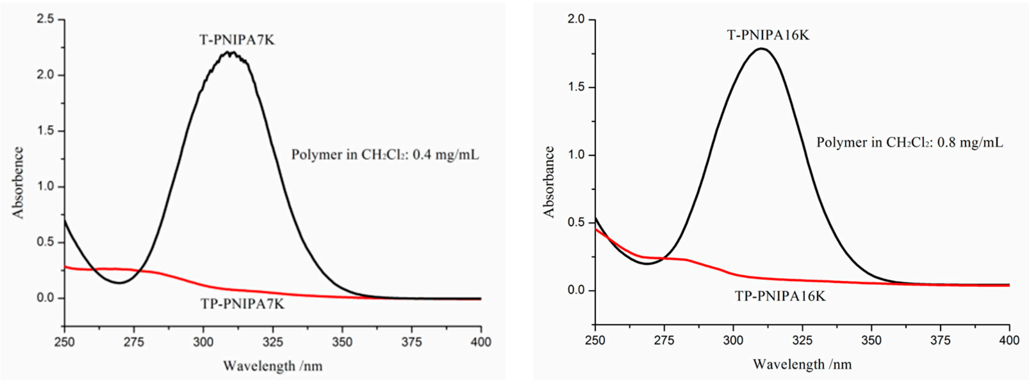

Figure S6. *Cont.*

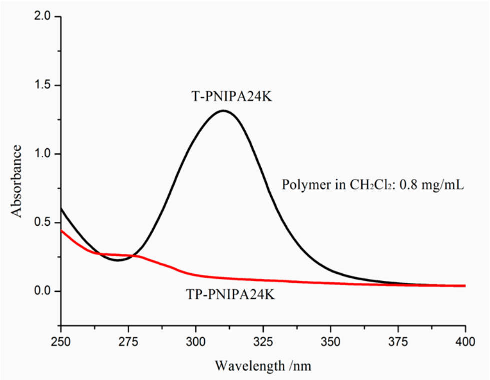

Figure S6. UV traces of PNIPAs before and after modification.

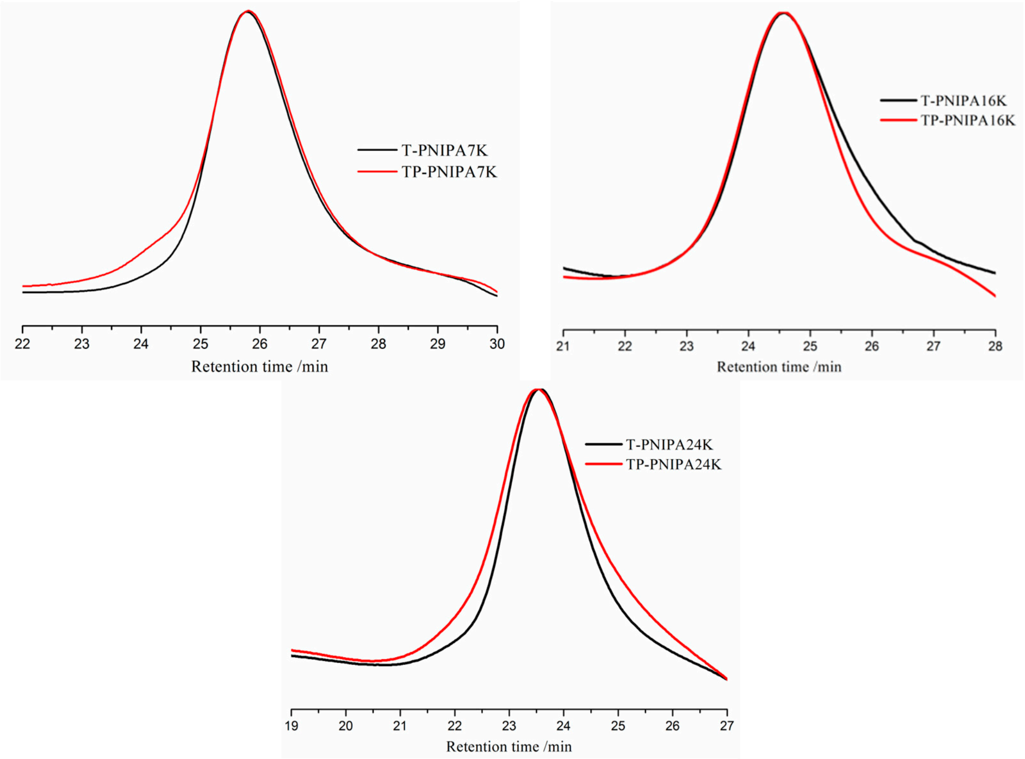

Figure S7. GPC traces of PNIPAs before and after modification.

© 2016 by the authors; licensee MDPI, Basel, Switzerland. This article is an open access article distributed under the terms and conditions of the Creative Commons by Attribution (CC-BY) license (http://creativecommons.org/licenses/by/4.0/).
